# Antipsychotic-Like Effect of Trimetazidine in a Rodent Model

**DOI:** 10.1155/2013/686304

**Published:** 2013-10-22

**Authors:** Oytun Erbaş, Hüseyin Serdar Akseki, Betül Eliküçük, Dilek Taşkıran

**Affiliations:** ^1^Gaziosmanpaşa University School of Medicine, Department of Physiology, 60100 Tokat, Turkey; ^2^Tavsanlı State Hospital, Department of Psychiatry, 43300 Kütahya, Turkey; ^3^Manisa Mental Health and Illnesses Hospital, 45000 Manisa, Turkey; ^4^Ege University School of Medicine, Department of Physiology, 35100 Izmir, Turkey

## Abstract

Trimetazidine (TMZ) has been used as an anti-ischemic agent for angina pectoris, chorioretinal disturbances, and vertigo. Also, it can induce extrapyramidal type adverse reaction such as parkinsonism, gait disorder, and tremor via blockade of D2 receptors. In the present study, we evaluated the effect of TMZ on novelty-induced rearing behavior and apomorphine-induced stereotypy behavior in male rats. Four groups of rat (*n* = 7) were administrated with TMZ (10 and 20 mg/kg, i.p.), chlorpromazine (1 mg/kg, i.p.), or isotonic saline. One hour later, apomorphine (2 mg/kg, s.c.) was administrated to each rat. Our results showed that both doses of TMZ significantly decreased the rearing behavior in rats, whereas the decrease with chlorpromazine was higher. TMZ also decreased the stereotypy scores in a dose-dependent manner. We concluded that TMZ has beneficial effects on rearing behavior and stereotypy, which are accepted to be indicators of antipsychotic effect. Taken together, with its antioxidative and cytoprotective properties, TMZ is worthy of being investigated for its anti-psychotic effects as a primary or an adjunctive drug.

## 1. Introduction

Schizophrenia is a psychiatric disorder, which is thought to have close relationship with hyperdopaminergic activity [1]. Based on pharmacological, as well as clinical, evidence, hyperdopaminergic activity in the mesolimbic pathway is believed to be responsible for the positive symptoms, whereas hypodopaminergic activity in the mesocortical pathway triggers cognitive impairment and affective symptoms [2, 3]. Drugs used in the treatment of the disease have the potential to cause extrapyramidal side effects by blocking D2 dopamine receptors mainly located in the striatum [4, 5].

 Majority of the studies reported a significant imbalance between oxidative stress levels and antioxidative enzyme activities in schizophrenia. For example, Pazvantoglu et al. [6] demonstrated that the severity of the symptoms was negatively correlated with the total antioxidant potentials, whereas Padurariu et al. [7] found conflicting results demonstrating the increased superoxide dismutase (SOD) activity and decreased glutathione peroxidase (GPx) activity in patients with schizophrenia compared to controls. However, the studies on the lipid peroxidation markers, such as malonyl dialdehyde (MDA) and 4-hydroxynonenal (4-HNE), showed more consistent results [7–10]. Wang et al. showed increased levels of 4-HNE in schizophrenic patients compared to normal controls [8]. Also, MDA levels have been found elevated in peripheral tissues of schizophrenic patients [7, 9]. 

 Trimetazidine (TMZ;  1-[2,3,4-trimethoxybenzyl]piperazine), an anti-ischemic drug, has been used in cardiology practice due to its protective effects particularly against myocardial ischemia and reperfusion injury [11–14]. Trimetazidine is thought to carry out its effects via supporting cellular homeostasis during ischemia-reperfusion injury. Both in vivo and ex vivo trials have revealed that TMZ reduces intracellular acidosis, regulates Na^+^ and Ca^2+^ levels, lessens membrane damage, preserves mitochondrial functions, regulates myocardial glucose utilization in favour of glucose use, and limits neutrophil infiltration in the ischemic area [15–19]. 

 For decades, TMZ has been demonstrated to be safe and effective in patients with coronary artery disease and neurosensory ischaemia. However, recent studies have reported that it can also induce extrapyramidal type adverse reaction such as parkinsonism, gait disorder, and tremor [20–22]. TMZ has the same piperazine core as cinnarizine and flunarizine, calcium channel blockers, which interacts with dopamine receptors (and particularly striatal dopamine D2 receptors) and leads to extrapyramidal disorders [20, 23].

Despite many studies depicting its extrapyramidal side effects, which are thought to be in close relationship with D2 dopamine receptor blockade, to date there are no preclinical studies about the antipsychotic-like effect of TMZ. Hence, in the present study, we hypothesized that TMZ may produce an antipsychotic-like effect in a rodent model for psychosis. In order to determine its efficacy in psychosis, we compared the effects of TMZ and chlorpromazine, a conventional anti-psychotic drug, by evaluating the novelty-induced rearing and apomorphine-induced stereotypic behavior in rats. 

## 2. Materials and Methods

### 2.1. Animals and Housing Conditions

 Twenty-eight adult male Sprague Dawley rats (220–240 g) were included in the study. All animals were kept under standard 12 h light/dark cycle in a temperature controlled (22 ± 2°C) environment with *ad libitum* access to rodent chow. All experimental procedures were performed during the light cycle (from 10:00 to 16:00). The experimental protocol performed in the study was approved by the Institutional Animal Care and Ethics Committee of the Ege University. 

### 2.2. Drugs

All drugs were freshly prepared. Apomorphine hydrochloride (Sigma Chemical Co., St. Louis, MO) was dissolved in saline containing 0.1% ascorbic acid prior to experiments. TMZ (Servier Drug Company, Istanbul, Turkey) was dissolved in saline. Saline (0.9% NaCl) was used as control solution. All solutions were administered intraperitoneally (i.p.) in a volume of 1 mL/kg body weight.

### 2.3. Assessment of Novelty-Induced Rearing Behavior

Novelty-induced rearing behavior is used to assess the central excitatory locomotor behavior in rodents [24]. Four groups of rat (*n* = 7) were administered TMZ (10 and 20 mg/kg, i.p.), chlorpromazine (1 mg/kg; i.p.), or isotonic NaCl (1 mL/kg, i.p.). One hour later, novelty-induced rearing behavior was explored by placing the animals directly from home cages to a transparent Plexiglas cage (45 cm × 25 cm × 25 cm). The rearing frequency (number of times the animal stood on its hind limbs or with its fore limbs against the walls of the observation box or free in the air) was recorded for 10 min. All rats were monitored individually by two observers who were blinded to the study groups. The arena was cleaned with 5% alcohol to eliminate olfactory bias before beginning a fresh animal.

### 2.4. Apomorphine-Induced Stereotypic Behavior Test

Mesolimbic and nigrostriatal dopaminergic pathways play crucial roles in the mediation of locomotor activity and stereotyped behavior. Apomorphine-induced stereotypy is due to the stimulation of dopamine receptors and has been used as a convenient method for *in vivo* screening of dopamine agonists or antagonists and assessment of dopaminergic activity [25, 26].

Briefly, four groups of rat (*n* = 7) were administered TMZ (10 and 20 mg/kg, i.p.), chlorpromazine (1 mg/kg, i.p.), and isotonic saline (1 mL/kg, i.p.). One hour later, apomorphine (2 mg/kg, s.c.) was administered to each rat. First, rats were placed into the cylindrical metal cages (18 × 19 cm) containing vertical (1 cm apart) and horizontal (4.5 cm apart) metal bars (2 mm) with upper lid for 10 minutes for orientation period. After apomorphine administration, the rats were immediately placed back into the metal cages and observed for stereotypic behavior. Signs of stereotypy, which include mainly sniffing and gnawing, were observed and scored as follows: absence of stereotypy (0), occasional sniffing (1), occasional sniffing with occasional gnawing (2), frequent gnawing (3), intense continuous gnawing (4), and intense gnawing and staying on the same spot (5). The stereotypic behavior was rated after each minute, and mean of 15 min period was calculated and recorded [27]. 

### 2.5. Statistical Analysis

 Statistical evaluation was performed by one-way analysis of variance (ANOVA). Post hoc Bonferroni test was used to identify differences between the experimental groups. Results are presented as mean ± SEM. A value of *P* < 0.05 was considered to be significant.

## 3. Results

### 3.1. The Effect of TMZ on Novelty-Induced Rearing Behavior

**Figure 1:**
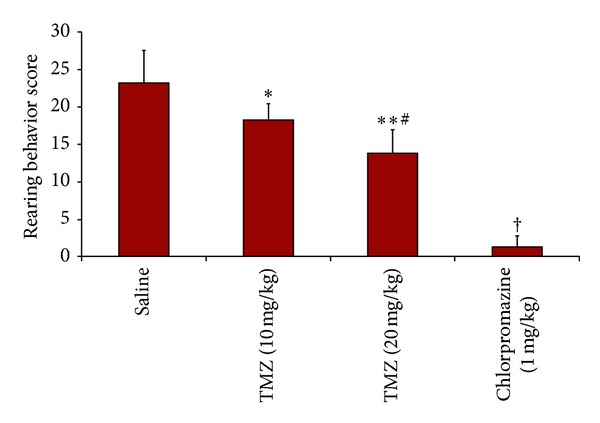
Rearing behavior scores. Data are expressed as mean ± SEM. Statistical analysis was performed by one-way analysis of variance (ANOVA) and Bonferroni's post hoc test. *different from saline,  *P* = 0.037, **different from saline,  *P* = 0.000, ^#^different from TMZ (10 mg/kg), *P* = 0.029, ^†^different from other groups, *P* = 0.000.

Figure 1 represents the effects of TMZ and chlorpromazine treatment on rearing behavior. ANOVA results revealed significant differences between the groups (*P* < 0.0005). Post-hoc Bonferroni test demonstrated a highly significant reduction in rearing behavior in TMZ (10 and 20 mg/kg) and chlorpromazine (1 mg/kg) administered rats compared to saline group (*P* = 0.037, *P* = 0.000, *P* = 0.000, resp.). The inhibitory effect of TMZ on rearing behavior was dose dependent, being more evident at a higher dose (20 mg/kg).

### 3.2. The Effect of TMZ on Apomorphine-Induced Stereotypic Behavior Test

**Figure 2:**
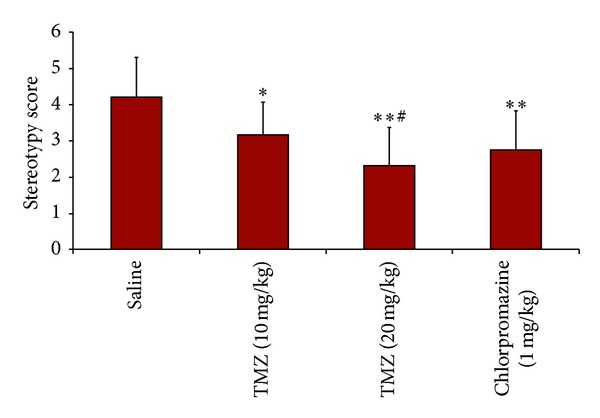
Apomorphine-induced stereotypy scores. Data are expressed as mean ± SEM. Statistical analysis was performed by one-way analysis of variance (ANOVA) and Bonferroni's post hoc test. *different from saline, *P* = 0.004, **different from saline,  *P* = 0.000, ^#^different from TMZ (10 mg/kg), *P* = 0.033.

Figure 2 depicts the effects of TMZ and chlorpromazine treatment on stereotypy scores. ANOVA results showed significant differences between the groups (*P* < 0.0005). Post-hoc Bonferonni test demonstrated a highly significant decrease in stereotypy scores in both doses of TMZ and chlorpromazine compared to saline group (*P* = 0.004, *P* = 0.000, *P* = 0.000, resp.). The decrease was significantly greater with 20 mg/kg of TMZ compared to 10 mg/kg (*P* = 0.033).

## 4. Discussion

This study demonstrates the beneficial effects of TMZ on rearing behavior and stereotypy, which are accepted to be indicators of anti-psychotic effect. Theoretically, anti-psychotic effect is mediated by means of antidopaminergic activity in certain regions of central nervous system. But adverse drug effects have brought a big burden for the patients. Therefore, clinical and nonclinical investigations focused on new drugs, which cause fewer side effects. 

Exposure of rodents to a new environment causes novelty-induced behavior syndrome consisting of rearing, grooming, and wet-dog shakes. The novelty-induced rearing behavior response is regulated by various neurotransmitter systems including GABA_A_, opioid, and dopamine D2 receptors [28]. In a previous study, Tejashree et al. examined the anti-psychotic-like effects of liraglutide, a GLP-1 agonist, and sitagliptin, which is a dipeptidyl peptidase (4DPP-4) inhibitor [29]. Liraglutide and sitagliptin are US FDA approved medications for the treatment of type 2 diabetes mellitus. Liraglutide treatment significantly attenuated apomorphine-induced cage climbing behavior, which is thought to be first preclinical evidence for anti-psychotic-like effect [29]. As TMZ reduces the utilization of fatty acids in favour of carbohydrates, it may share a common mechanism with liraglutide in the cellular level. In Tejashree's study, liraglutide showed equal effect with haloperidol in reversing apomorphine-induced cage climbing behavior. In the present study, TMZ decreased rearing behavior in rats in a dose-related manner, which is also reduced by chlorpromazine more efficiently. Chlorpromazine, a very effective antagonist of D2 dopamine receptors, exerts additional antiadrenergic, anticholinergic, and antihistaminergic effects [30]. Hence, the efficacy of chlorpromazine on rearing behavior may be associated with its sedative effect, which is mainly maintained by anticholinergic and antihistaminergic properties of that drug. 

 Sotoing Taïwe et al. [28] examined the effect of aqueous extract and alkaloid fraction of *Crassocephalum bauchiense* in rodents. Both aqueous extract and the alkaloid fraction caused dose-dependent inhibition in the rearing behavior, which is mediated through GABA-A, opioid, and D2 dopamine receptors [28]. Stereotypical behavior is a common feature manifested in schizophrenia and is increased by apomorphine probably through D2 receptors. Besides rearing behavior, Sotoing Taïwe et al. showed that aqueous extract and alkaloid fraction of *Crassocephalum bauchiense* decreased the apomorphine-induced stereotypy scores [28]. In our study, TMZ also significantly lessened the stereotypy scores.

 Recently, Masmoudi et al. [20] reported the series of 21 cases that had extrapyramidal disorders associated with TMZ use. The TMZ-associated drug reactions were typical parkinsonism, gait disorders, and restless leg syndrome. The discontinuation of the drug led to total disappearance of symptoms in 16 patients [20]. Similarly, Bondon-Guitton et al. [21] reviewed drug-induced or drug-worsened parkinsonism cases reported to a pharmacovigilance center between 1993 and 2009 and reported three notifications with TMZ. Since TMZ owns a piperazine core in the chemical formula as in flunarizine, it can blockade D2 dopamine receptors and lead to extrapyramidal type adverse reaction [20, 22].

 In recent years, glutamate has been paid much attention because of its role in schizophrenia. Glutamatergic dysfunction is thought to be one of the possible etiologic factors in schizophrenia. It is known that glutamatergic transmission is primarily mediated through its metabolic and ionotropic receptors. The ionotropic receptors are N-methyl-D-aspartate (NMDA), alpha-amino-3-hydroxy-5-methyl-4-isoxazolepropionic acid (AMPA), and kainate receptors. Hypofunctional NMDA receptors are accused of being responsible in the pathogenesis of schizophrenia [30–32]. In a postmortem study, Noga et al. [33] reported increased AMPA binding in striatal structures such as caudate, putamen, and accumbens in schizophrenia using [^3^H]CNQX. Similarly, Zavitsanou et al. [34] found increased [^3^H]AMPA binding in the superficial layers of cortex which suggests a postsynaptic compensation for impaired glutamatergic neurotransmission in schizophrenia. On the other hand, Dayanithi et al. [35] investigated the effects of TMZ on AMPA and kainate receptors in rat vestibular ganglion neurons, and they found that TMZ could be a potent antagonist of AMPA and kainate receptors. This study suggests that TMZ may have modulatory effects on non-NMDA glutamatergic receptors, which are thought to play an important role in schizophrenia.

 It is well known that anti-psychotic drugs are the first choices for the treatment of schizophrenia. Majority of the studies indicated the increased levels of oxidative stress parameters after the treatment with classical antipsychotics. For example, Sagara et al. [36] showed that haloperidol induced a sixfold increase in levels of reactive oxygen species (ROS) and treatment of antioxidants, such as vitamin E, lowered the levels of ROS, and protected the cells. Similarly, Reinke et al. [37] revealed that haloperidol and clozapine were related with oxidative stress in the rat brain, but haloperidol-receiving group showed a higher increase compared to clozapine. More recently, Kropp et al. [38] measured the MDA levels in schizophrenic patients during treatment with first- and second-generation antipsychotics. According to their results, MDA levels in patients receiving clozapine, quetiapine, and risperidone were lower than the first-generation antipsychotic receiving group. They found that atypical anti-psychotics attenuated the oxidative stress and decreased oxidative damage markers. On the other hand, it has been claimed that increased oxidative stress seen in some clozapine treated patients could be related to the illness severity since clozapine is mainly used in refractory patients [39]. 

All these studies point out that there is a growing body of evidence proving the importance of oxidative stress in schizophrenia both in pathogenesis and treatment modalities. So, it is suggested that antioxidants might be useful in the treatment of schizophrenia. For instance, Dakhale et al. [40] indicated that vitamin C and oral anti-psychotic combination reduced brief psychiatric rating scale scores and MDA levels. Zhang et al. showed that Ginkgo biloba extract (a powerful antioxidant) and haloperidol combination resulted in better positive and negative syndrome scale (PANSS) scores and reduced extrapyramidal side effects [41, 42]. In a meta-analysis, Singh et al. [43] reported that Ginkgo biloba combined with anti-psychotics exhibited improvement in psychotic symptoms. As the majority of the studies show the improving effects of anti-oxidants as an adjunct therapy, we propose that TMZ might be beneficial with its obvious anti-oxidant effects in schizophrenia.

## 5. Conclusion

Considering the effects of TMZ on rearing behavior and stereotypical behavior in rats, we propose that TMZ may have anti-psychotic-like potential because of its antidopaminergic effects. On the other hand, TMZ, as an anti-oxidant and cytoprotective agent, can be useful in neuroprotection especially on early stages of psychosis or prepsychotic patients with insignificant symptoms. In addition, as glutamatergic excitotoxicity is responsible for the neurodegeneration and neuron loss, which is possibly related with negative symptoms and cognitive dysfunction, TMZ might be having a potential as a regulator on glutamatergic system. However, as we have little data about trimetazidine's anti-dopaminergic effects and glutamatergic modulating roles compared to its well-known anti-oxidant effects in psychotic patients, this study needs to be supported by further experimental and clinical research.
